# Flavanols and Anthocyanins in Cardiovascular Health: A Review of Current Evidence

**DOI:** 10.3390/ijms11041679

**Published:** 2010-04-13

**Authors:** Sonia de Pascual-Teresa, Diego A. Moreno, Cristina García-Viguera

**Affiliations:** 1 Department of Metabolism & Nutrition, Instituto del Frío, CSIC, Madrid E-28040, Spain; E-Mail: soniapt@if.csic.es; 2 Department of Food Science and Technology, Centro de Edafología y Biología Aplicada del Segura (CEBAS), CSIC, Murcia E-30100, Spain; E-Mail: cgviguera@cebas.csic.es

**Keywords:** anthocyanin, cardiovascular, catechin, flavonoids, proanthocyanindins

## Abstract

Nowadays it is accepted that natural flavonoids present in fruits and plant-derived-foods are relevant, not only for technological reasons and organoleptic properties, but also because of their potential health-promoting effects, as suggested by the available experimental and epidemiological evidence. The beneficial biological effects of these food bioactives may be driven by two of their characteristic properties: their affinity for proteins and their antioxidant activity. Over the last 15 years, numerous publications have demonstrated that besides their *in vitro* antioxidant capacity, certain phenolic compounds, such as anthocyanins, catechins, proanthocyanidins, and other non coloured flavonoids, may regulate different signaling pathways involved in cell survival, growth and differentiation. In this review we will update the knowledge on the cardiovascular effects of anthocyanins, catechins and proanthocyanidins, as implied by the *in vitro* and clinical studies on these compounds. We also review the available information on the structure, distribution and bioavailability of flavanols (monomeric catechins and proanthocyanidins) and anthocyanins, data necessary in order to understand their role in reducing risk factors and preventing cardiovascular health problems through different aspects of their bioefficacy on vascular parameters (platelet agregation, atherosclerosis, blood pressure, antioxidant status, inflammation-related markers, *etc.*), myocardial conditions, and whole-body metabolism (serum biochemistry, lipid profile), highlighting the need for better-designed clinical studies to improve the current knowledge on the potential health benefits of these flavonoids to cardiovascular and metabolic health.

## 1. Introduction

**Figure 1.**
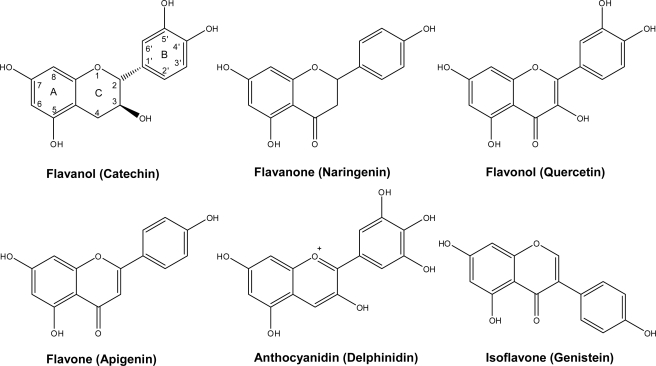
General structure of common flavonoids in plant-derived foodstuffs.

Flavonoids occur widely in the fruits and vegetables that make up the human diet and it has been estimated that at least one gram of flavonoid is daily consumed [1,2]. Whether this is beneficial to human health is still debatable [2]. Flavonoids include a series of subclasses with a common general structure characterized by the presence of two aromatic rings linked by a three-carbon bridge [1–4]. These subclasses, classified based on the connection between the two aromatic rings, as well as the oxidation state of the different rings, are: flavanols [e.g., monomeric (catechin, epicatechin), oligomeric, and polymeric compounds (proanthocyanidins, also called condensed tannins)] typically found in tea, cocoa, grape and wine; flavanones (*i.e.*, hesperetin, found in citrus); flavones (*i.e.*, luteolin; in thyme, rosemary and oregano); isoflavones (*i.e.*, genistein, in soybean); flavonols (*i.e.*, quercetin, in onions and most fruits and vegetables); and anthocyanins (*i.e.*, cyanidin, in red, blue and purple berries) [1–4] (Figure 1).

Flavonoids fall into two major categories, according to whether the central heterocyclic ring is unsaturated or not. When unsaturation is present, as in the anthocyanins, flavones and flavonols, the molecule is planar. This planarity may occasionally be distorted, e.g., by the substitution of the 2′-hydroxyl in a 3-*O*-methylflavonol, causing a bathochromic shift in the spectral properties. Saturated flavonoids (flavanones, flavans) have one or more chiral centres, and can thus exist in more than one optically active form. In fact, they generally take up a conformation with the two benzene rings at right angles. Optical activity may also be present in flavonoids due to glycosidic substituents [1–3].

Cardiovascular disease (CVD) is caused by disorders of the heart and blood vessels, and includes coronary heart disease (heart attacks), cerebrovascular disease (stroke), raised blood pressure (hypertension), peripheral artery disease, rheumatic heart disease, congenital heart disease and heart failure. The major causes of cardiovascular disease are consumption of tobacco, physical inactivity, and an unhealthy diet [5]. An estimated 17.1 million people died from cardiovascular diseases (CVDs) in 2004, representing 29% of all global deaths. By 2030, almost 23.6 million people will die from CVDs, mainly from heart disease and stroke [4,5].

Flavonoids contribute to different extents to the beneficial health effects of a diet rich in fruits and vegetables. Their actions are potentially beneficial in a wide range of diseases, from cardiovascular disease to cancer and neurodegenerative conditions. Many studies have shown the antioxidant power of particular flavonoids and flavonoid-rich extracts. However, nowadays, it is widely accepted that if flavonoids have any preventive or curative activity through their ingestion, this effect must involve, not only their antioxidant potential, but also the modulation of multiple cellular pathways that are crucial in the pathogenesis of those diseases [3,4].

Certain plant-derived foods and drinks, including chocolate, wine, berry juices, different types of teas, *etc.*, have been targeted for different studies in the last decades for their potential use or benefit on cardiovascular health, both *in vitro* and *in vivo*. These foods contain a wide range of phenolic compounds, including flavonols, flavanols (catechins), procyanidins and/or anthocyanins. In the present work, we have reviewed what is known on flavanols (monomeric and polymeric compounds) and anthocyanins, including their distribution in foods, their bioavailability and their potential influence on cardiovascular problems or their cardiovascular health-promoting effects, as well as the possible molecular mechanisms underlying this effect.

Other flavonoids not cited in this work may also be involved in the positive cardiovascular health effect of fruits and vegetables, however we consider that these other flavonoid subclasses have already been reviewed in depth by other authors [6–9].

## 2. Chemistry and Distribution

### 2.1. Flavanols: Monomeric Catechins and Proanthocyanidins

**Figure 2.**
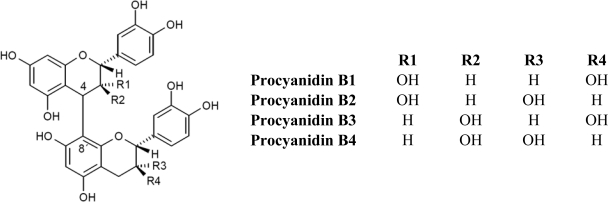
Chemical structure of common oligomers of the B-type procyanidins (B1 to B4).

Monomeric flavanols or catechins (flavan-3-ols), biosynthetic precursors of proanthocyanidins, are characterized for having a C_6_-C_3_-C_6_ skeleton with a hydroxyl group in position three of the C-ring (Figure 1). Flavan-3-ols represent the largest class of monomeric C_6_-C_3_-C_6_ flavanols. The two compounds catechin and epicatechin are among the commonest flavonoids known, sharing a distribution almost as widespread as the related flavonol, quercetin [1,2]. Catechin (C), epicatechin (EC), gallocatechin (EC), epigallocatechin (EGC) and their galloyl substituted derivatives (ECG, and EGCG) are usually found in plant-derived foods and food products. Catechins are rarely found in nature in their glycosylated form, unlike anthocyanidins which are commonly found as anthocyanins. On the other hand, flavanols are commonly found in plant-derived food products in their polymerized forms as oligomers (dimers to pentamers) or polymers (six or more units). The most common oligomers are the B series, B1 to B8, formed by two flavanol units, either catechin or epicatechin, joined by a C4–C8 linkage (B1 to B4) [4→8 bond (epicatechin-(4β→8)-catechin); Figure 2], or C4–C6 linkage (B5 to B8). The least frequent dimers are the A series, characterized by the presence of double linkages between the two “catechin” units, one C4–C8 or C4–C6 and an additional one between C2 and C5 or C7 [1,2].

**Figure 3.**
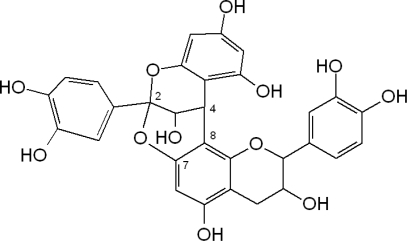
Chemical structure of dimeric type-A proanthocyanidin.

Type-A proanthocyanidins (Figure 3) are less common in food plants, however their presence in peanuts and almond skins and in some berries has been described [9–12]. In general, flavanols are widely distributed in plant foods.

Flavanols are mainly present in fruits and derived products like fruit juices or jams; also in tea, cocoa, and cereals (Table 1). They are however almost non-existent in vegetables and legumes, with the notable exception of lentils and broad beans [12,13]. In many cases flavanols are present in the peels or seeds of fruits and vegetables, being discarded when eaten or during processing, and therefore their dietary intake is limited.

Consumption of total catechins and dimeric procyanidins has been estimated to be between 18 and 50 mg per day in the Spanish population [26] and 50 mg (s.d. 56 mg/day) catechins per day in a nationwide dietary survey among 6,200 Dutch men and women aged 1–97 years [27]. Catechin intake increased with age, and the intake was higher in women (60 mg/day) than in men (40 mg/day) [27]. However it has to be taken into consideration that the bioavailability of flavanols varies depending on the degree of polymerization and the existence of galloyl residues in the molecule.

The food sources of catechins (tea, chocolate, apples, pears, grapes and red wine) are very popular and highly consumed. The usual sources of dietary catechins in the studies of bioavailability of flavanols with human volunteers are cocoa or tea [28]. After ingestion of chocolate, representing doses of 46, 92, and 138 mg of unmetabolized catechin, the maximal plasma concentrations (*C_max_*) of 0.13, 0.26, and 0.36 μmol/L, respectively, were reached in 2 h (*T_max_*) [26]. Similarly, 220 mg of epicatechin (EC) from chocolate ingestion reached *C_max_* of 4.77 μmol/L also in 2 hours, with urinary excretion (as a % of intake) of 29.8% [28]. Tea catechins (mainly constituted by epigallocatechin and epigallocatechin-3-gallate) at a total dose of 240 mg gave a 0.5 μmol/L *C_max_* with a *T_max_* that, depending on the specific metabolites, ranged from 1.6 to 2.3 hours, with a mean level of excretion of 8.1 (% of intake), but with clear differences between catechins (28.5%) and gallocatechins (11.4%) [29].

Bioavailability differs markedly among catechins, and galloylation of catechins also reduces their absorption. The wide nature of procyanidins and flavan-3-ols affects their bioavailability since they can be detected at different degrees of polymerisation and acylation (with gallic acid). The galloylation of flavan-3-ols reduces their absorption; polymerised procyanidins are not absorbed and small amounts of procyanidin dimers B1 and B2 have been detected in plasma [30–32]. Galloylated catechins are rapidly eliminated, probably not by degalloylation, which has been shown to be a minor process in humans, but rather by preferential excretion of these compounds in bile [27,28]. In all cases, catechin and gallocatechin were found in urine excreted as methylated and/or sulphated or glucuronidated forms. We should note that, as stated by Stalmach *et al.* [29], there is still a controversy regarding the bioavailability of catechins, mainly due to a lack of a standardised methodology for both the processing of plasma samples and the analytical determination of the metabolites in the processed extracts.

The maximum concentration of metabolites of flavan-3-ols in plasma varied between 0.03 and 2.7 μmol/L (after a 50 mg aglycone equivalent supply) and the urinary excretion was 0.1–55%. The high interval in the absorption and urinary excretion was due to the wide variability in the nature of flavan-3-ols [28].

Epigallocatechin gallate is the only known polyphenol present in plasma in large proportions in a free from. The flavan-3-ols are highly methylated and glucuronidated in the 3′ and 4′ positions (catechin and epicatechin metabolites mainly) [33].

Procyanidin intake in the United States, excluding monomers, has been estimated to be 53.6 mg per day [34]. Very few studies have shown absorption of procyanidin dimers in humans [32–37] and none has shown, so far, absorption of higher polymerized compounds in humans. However certain studies have reported the absorption of procyanidins, up to trimers, in rats [38,39], showing that in the case of flavanol dimers and trimers, no glucuronidation or sulphatation take place.

The flavanols is a very complex group of polyphenols ranging from the monomeric flavan-3-ols (catechin and epicatechin; gallocatechin; and epigallocatechin and the corresponding gallate esters) to polymeric procyanidins known as condensed tannins including a whole range of oligomeric intermediates going from dimers up to undecamers and dodecamers and then polymers, mainly provided by fruits, tea and wine. The main products or metabolites detected in urine as a result of the bacterial metabolism after the intake of catechin and epicatechin are 3-hydroxyphenylpropionic acid, δ-(3,4,-dihydroxyphenyl)-γ-valerolactone; δ-(3-hydroxyphenyl)-γ–valerolactone; 3-hydroxyhippuric acid (from both bacterial and human metabolism) [40–43].

After *in vitro* incubation of epicatechin with human intestinal bacteria the metabolites pyrogallol, 3′,4′-dihydroxyphenylvaleric acid, 3′-hydroxyphenylvaleric acid, 3,4-dihydroxyphenylpropionic-, 3′-hydroxyphenylpropionic-, 3′-methoxyphenylvaleric acid and 2″,3″-dihydroxyphenoxyl-3′,4′-dihydroxyphenylpropionic acid were detected. However, epicatechin gallate and epigallocatechin gallate were not degraded by the microbiota [44].

The quantitative importance and biological activities of the microbial metabolites have seldom been examined *in vivo* by measuring the microbial metabolites formed. In four groups of rats fed for 8 days a diet supplemented with 0.12 g/100 g catechin, 0.25 or 0.5 g/100 g red wine powder containing proanthocyanidins, phenolic acids, flavanols, anthocyanins and flavonols or an unsupplemented diet the main metabolites from the catechin diet were 3-hydroxyphenylpropionic acid, 3-hydroxybenzoic acid and 3-hydroxyhippuric acid. Their total urinary excretion accounted for 4.7 g/100 g of the catechins ingested and that of intact catechins for 45.3 g/100 g. For wine polyphenols, the same microbial metabolites as observed for the catechin diet were identified in urine, along with hippuric, *p*-coumaric, vanillic, 4-hydroxybenzoic and 3-hydroxyphenylacetic acid. All together, these aromatic acids accounted for 9.2 g/100 g of the total wine polyphenols ingested and intact catechins for only 1.2 g/100 g. The higher excretion of aromatic acids by rats fed wine polyphenols is likely due to their poor absorption in the proximal part of the gut. Since microbial metabolites still bear a reducing phenolic group and should also prevent oxidative stress in inner tissues, these microbial metabolites and their biological properties help explain why the health effects of polyphenols deserve more attention [45].

Studies on the microbial metabolism of dietary condensed tannins (oligomeric and polymeric procyanidins) showed that benzoic, phenylacetic, phenylpropionic and phenyllactic acid derivatives, as well as phloroglucinol, δ-(3-hydroxyphenyl)-γ-valerolactone, and 1-(3-hydroxyphenyl)-3-(2,4,6-trihydroxyphenyl) propan-2-ol were produced in rat cecal models [46]. In another study, condensed tannins produced 4-hydroxy-phenylacetic, 3-phenylpropionic, 3-hydroxyphenylacetic, 4-hydroxyphenylpropionic, 3-hydroxyphenylvaleric and 3-hydroxyphenyl propionic [41], while valerolactones were not detected.

As instestinal metabolism strongly influences the bioavailability of flavonoids, the investigated microbial deconjugation and degradation of the most common flavan-3-ols using the pig cecum *in vitro* model system developed by Van’t Slot and Humpf [47], showed that the microbial degradation of (+)-/(−)-catechin, (−)-epicatechin, (−)-gallocatechin, (−)-epigallocatechin, (−)-gallocatechin gallate, (−)-epigallocatechin gallate, procyanidin B2, and gallic acid, under anaerobic physiological conditions in single incubation experiments and as a mixture, was complete within 4–8 h. No difference was observed for catechin enantiomers. In addition to monomeric flavonoids, procyanidins were also metabolized by the intestinal microbiota, as shown for procyanidin B2. The hydroxylated phenolcarboxylic acids produced were similar for all the tested substances [47].

### 2.2. Anthocyanins

Anthocyanins are natural pigments responsible for the blue, purple, red and orange colors of many fruits and vegetables. More than 500 different anthocyanins have been described in the literature. Anthocyanins are food bioactive compounds with a double interest, one technological, due to their impact on the sensorial characteristics of food products, and the other for their health related properties throw different biological activities, one of them being their implication on cardiovascular disease risk protection [48].

**Figure 4.**
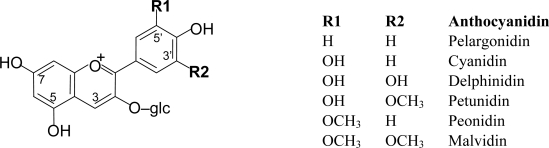
Structures of the most frequently found anthocyanidins in plant-derived foods.

Anthocyanins are mainly present in nature in the form of heterosides. The aglycone form of the anthocyanins, so-called anthocyanidin, is structurally based on the flavilium or 2-phenyl-benzopyrilium cation, with hydroxyl and methoxyl groups present at different positions of the basic structure (Figure 4). Depending on the number and position of the hydroxyl and methoxyl groups as substituents, different anthocyanins have been described, and six of them are commonly found in fruits and vegetables: pelargonidin, cyanidin, delphinidin, petunidin, peonidin and malvidin [2,3,13].

Anthocyanidins are unstable to light and are water-insoluble so that they do not occur usually in their free state. Instead, they are present in the cell vacuole linked to sugars, which provide stability and water solubility. These glycosides are called anthocyanins. Some time ago, the known glycosidic variation among these pigments was restricted to four main types: 3-monoglycosides, 3-diglycosides, 3,5-diglycosides and 3-diglycoside-5-monoglycosides. The most common sugar present was glucose, but rhamnose, xylose and galactose were also encountered. More recent research has revealed many more complex structures. There are many anthocyanins with acyl substituents linked to sugars, aliphatic acids (such as malonic, succinic, malic and acetic acid), cinnamic acids (such as *p*-coumaric, ferulic, or sinapic acid), and pigments with both aliphatic and aromatic substituents. A further complexity in some anthocyanins results from the presence of several acylated sugars in the structure. These anthocyanins have sometimes been designated as polyglycosides [2,3,13,49].

The main dietary sources of anthocyanins are red fruits (Table 2), like berries and red grapes, red wine, cereals and purple corn, as well as some vegetables such as red cabbage [48–73]. Daily consumption of total anthocyanins has been estimated to be between 3 and 215 mg/day [55,71,76,77]. Since dietary anthocyanins are restricted to berries, red fruits and red wine it should be assumed that there are variations between individuals due to differences in the intake of these anthocyanin-rich products. However, the influence of methodological differences in the assessment, as well as nutritional, social and cultural differences of the investigated populations may also explain the wide range of anthocyanin consumption estimated by different authors [28,48,49].

Until very recently, anthocyanins were believed to have a very low bioavailability. Clinical studies carried out with humans consuming different types of fruit containing anthocyanins have shown that these phenolic pigments are poorly absorbed [78,79]. The concentration of anthocyanins in plasma ranged 10–50 nmol/L (after a 50 mg of aglycone equivalent supply) and the *T_max_* was 1.5 h. At intakes of 188–3,570 mg total cyanidin glycosides, the *C_max_* were in the range of 2.3–96 nmol/L [71,81]. The urinary excretions were found to be in the range between 0.018% and 0.37 % of the intake. The time of appearance in the plasma was consistent with absorption at the stomach (24%) but also at the small intestine (23%) [74,82–84]. Maximal urinary excretion is usually achieved in less than 4 h. Nevertheless, some studies revealed that the bioavailability of anthocyanins was underestimated since all of their metabolites might not have been yet identified [28].

Anthocyanins are some of the few polyphenols that can be detected in plasma in the native form found in plant foods (glycosides). However, they can also suffer glucuronidation and sulphatation reactions as glucuronide and sulphate conjugates were detected in urine in several studies. Kay and co-workers [85] have also identified an oxidized derivative of anthocyanins. Methylation at the 3′-hydroxyl is also very common in anthocyanins [82,86]. The ratio of metabolites present in plasma or urine varied depending on the experiment, type of food administered, sample conservation, extraction procedures as well as the sensitivity and resolution of the HPLC methods used (6% [87], 25% [88], 68% [85], or 85% [82]).

Besides the usual organs where the metabolization and elimination of the anthocyanins takes place, like liver or kidney, these polyphenols have also been detected, in the course of animal studies, in brain regions that mediate cognitive behaviour (cortex, hippocampus), and eye [74,90,91].

Only a small part of the dietary anthocyanins are absorbed as such or as hydrolysis products in which the sugar moiety is removed. Thus, large amounts of the ingested compounds are likely to enter the colon. The studies looking at the colonic microbiota metabolism of anthocyanins are scarce. Using *in vitro* models, the microbial deglycosylation and degradation of six anthocyanins exhibiting three different aglycones with mono- or di-β-d-glycosidic bonds were investigated using HPLC-DAD and GC-MS detection [92]. All the anthocyanidin glycosides detected were hydrolysed by the microflora within 20 min and 2 h of incubation, depending on the sugar moiety. Due to the high instability of the liberated aglycones at neutral pH, primary phenolic degradation products were already detected after 20 min of incubation. Further metabolism of the phenolic acids was accompanied by demethylation. Because of their higher chemical and microbial stability, phenolic acids and/or other anthocyanin metabolites might be mainly responsible for the observed antioxidant activities and other physiological effects *in vivo* [92]. Cyanidin 3-rutinoside is first transformed into the corresponding glucoside and then to the aglycone. This is reported to be achieved by gut microbiota, and the aglycones, which are chemically unstable, are converted to phenolic acids that then can be further metabolized by the gut microbiota. The anthocyanin nucleus is broken down and protocatechuic (3,4-dihydroxybenzoic acid) acid is detected as a product of human colonic microbiota [92,93]. Other metabolites produced *in vitro* by the pig gut microbiota include syringic acid (3,5-dimethoxy-4-hydroxybenzoic acid), vanillic acid (3-methoxy-4-hydroxybenzoic acid), phloroglucinol aldehyde (2,4,6-trihydroxybenzaldehyde), phloroglucinol acid (2,4,6-trihydroxybenzoic acid), and gallic acid (3,4,5-trihydroxybenzoic acid), depending on the chemical structure of the anthocyanins [92]. In the case of methylated anthocyanins, the ring-cleavage degradation products can be de-methylated by the pig gut microbiota. This shows that anthocyanins as being flavonoid compounds, also suffer the ring cleavage, to release the B-ring and A-ring derived products [74].

More recently, it has been shown that anthocyanins may be metabolised by intestinal microflora [94,95], or simply being chemically degraded [85], producing a set of new products that have not yet been totally identified, but that include the phenolic acids: gallic acid, protocatechuic acid, syringic acid, vanillic acid and phloroglucinol. Looking only to the anthocyanin glucuronides and sulphates, the existent studies on anthocyanin bioavailability in humans have shown that for a total anthocyanin intake of 0.05–1.9 g, the corresponding *C_max_* of 0.001–0.2 μM was reached (*T_max_*) in a time range of 0.5–4 hours after ingestion of the respective dose, with a urinary excretion that in no case exceeded 5% of the ingested dose [96].

## 3. Cardiovascular Effects

### 3.1. Flavanols: Monomeric Catechins And (Oligomeric) Proanthocyanidins

#### 3.1.1. Catechins

There are a large number of reports on the *in vitro* effects of individual phenolics or plant extracts on various aspects of human health. However, many of these investigations lack any physiological significance because of the high doses used, typically of parent compounds such as flavonoids, rather than their conjugated mammalian metabolites or microbial degradation products [8,74]. Catechin and proanthocyanidins have proved to be potent antioxidants in different *in vitro* models, and in some *in vivo* intervention studies. However their potential beneficial effect on cardiovascular health is not merely due to this property but includes the different mechanisms implicated on cardiovascular conditions or problems, *i.e.*, hypertension, inflammation, cellular proliferation, trombogenesis, hyperglycaemia and hypercholesterolemia.

Platelet activation and subsequent aggregation play a major role in the pathogenesis of myocardial infarction and ischaemic heart disease. Hence, promoting an optimal platelet function *via* the reduction of platelet hyper-reactivity using dietary solutions is considered an interesting approach for the maintenance of cardiovascular health. Early *in vitro* studies using anesthetized dogs in a model of thrombosis and arterial damage showed that the naturally occurring compounds in red wine and purple-grape juice abolished the cyclic flow reductions in coronary blood flow. The biological activity of these compounds could explain the platelet-inhibitory properties of red wine and grape juice independently of the presence of alcohol [97].

The major catechin in green tea, epigallocatechin gallate (EGCG), decreases vascular inflammation by increasing the synthesis of nitric oxide (NO), which blocks endothelial exocytosis, the initial step in leukocyte trafficking and vascular inflammation [98]. EGCG decreased the activity and protein levels of inducible NO synthase (iNOS) by reducing the expression of iNOS mRNA and the reduction could occur through prevention of the binding of nuclear factor-κβ (NF-κβ) to the iNOS promoter, thereby inhibiting the induction of iNOS transcription [99].

Peroxisome proliferator-activated receptor β/δ (PPAR-β/δ) is selectively activated by green tea catechins (mainly EGCG), representing a key event in the reduction of NO production by green tea [100]. In hemodialysis patients, the chronic administration (3^th^ and 5^th^ month of a seven month study) of decaffeinated green tea extracts (catechins tablet; 455 mg/d comparable to four cups of green tea) decreased atherosclerotic factors such as tumor necrosis factor (TNF-α), soluble intercellular adhesion molecule 1 (sICAM-1), monocyte chemoattractant protein 1 (MCP-1), and C reactive protein (CRP) with respect to the control group receiving placebo, and authors concluded that the supplementation with decaffeinated green tea extracts (catechins) could be effective in reducing the levels of hemodialysis-induced reactive oxigen species (ROS) and palliating the subsequent adverse events– atherosclerosis and proinflammation [101].

In healthy human subjects, the ingestion of flavanol-rich cocoa (821 mg of flavanols/day, quantitated as (−)-epicatechin, (+)-catechin, and related procyanidin oligomers) induces vasodilatation via activation of the NO system, providing a plausible mechanism for the protection that flavanol-rich foods induce against coronary events [102].

Individual catechins isolated from green tea were investigated because of their effect on myocardial and whole-body blood pressure and it was demonstrated that each catechin (for example, EGCG), like a NO donor, may have a therapeutic use as an NO-mediated vasorelaxant and may have an additional protective action in myocardial ischemia-reperfusion induced injury. Epigallocatechin gallate (EGCG) modulates myocardial contractility showing Ca^2+^-dependent positive inotropic and lusitropic effects that are mediated in part via activation of the Na^+^/H^+^ transporter and the reverse mode of the Na^+^/Ca^2+^ transporter in the rat myocardium [103]. Cardiac hypertrophy is a pathological response of the heart to chronic pressure or volume overload, and is an independent risk factor for ischemic heart disease, arrhytmia, and sudden death. Green tea EGCG at 50 and 100 mg/L inhibits cardiomyocyte apoptosis–a critical factor during the transition from compensatory hypertrophy to heart failure–from oxidative stress *in vitro* and the molecular mechanisms implicated might be related to the inhibitory effects of EGCG on p53 induction and B-cell lymphoma protein 2 (bcl-2) decrease [104].

Cocoa polyphenols (flavonols and procyanidins) may exhibit antioxidant and anti-inflammatory, as well as anti-atherogenic activity. Several molecular targets (e.g., NF-κB, iNOS, angiotensin converting enzyme) have been recently identified from the *in vitro* cell culture experiments and *in vivo* animal studies together with the human intervention trials, proving that flavanols and procyanidins may partly explain potential beneficial cardiovascular effects of cocoa, either in powder, as chocolate or from different cocoa flavanol-rich drinks [105].

Obese or near-obese Japanese children were recruited for a double-blind, randomized, controlled study. The subjects ingested green tea containing 576 mg catechins, once per day for 24 weeks. The catechin group presented a decrease in waist circumference, systolic blood pressure, and low-density lipoprotein cholesterol (LDL) in obese or pre-obese children [106] and epigallocatechin gallate reduce diastolic blood pressure in overweight or obese adults [107]. The findings suggest that ingestion of a catechin-rich beverage ameliorates serious obesity and cardiovascular disease risk factors without raising any safety concerns in Japanese children [106].

Treatment with green tea extract for five weeks produced a significant 37.4% reduction in the concentration of oxidized LDL, and a highly significant increase in the mean diameter of the brachial artery following the post-compression hyperaemia phase in healthy women, producing modifications in vascular function and an important decrease in serum oxidizability [108,109].

The role of flavonoids in CVD, especially in strokes, is unclear. Mursu *et al.* [110], studied the association between the intakes of five subclasses (flavonols, flavones, flavanones, flavan-3-ols, and anthocyanidins), a total of 26 flavonoids, on the risk of ischaemic stroke and CVD mortality, in a study population of 1,950 eastern Finnish men aged 42–60 years free of prior cardiovascular health disorders or stroke (The Kuopio Ischaemic Heart Disease Risk Factor Study), and during an average follow-up time of 15.2 years, 102 ischaemic strokes and 153 CVD deaths occurred. The men in the highest quartile of flavonol and flavan-3-ol intakes had a relative risk of 0.55 and 0.59 for ischaemic stroke, respectively, as compared with the lowest quartile. The high intakes of flavonoids may be associated with decreased risk of ischaemic stroke and possibly with reduced CVD mortality. Additionally, the reduction of the relative risk for CVDs observed after flavonoid intake is clinically significant [110]. The reduction of systolic blood pressure by 5.9 mmHg after chronic intake of chocolate or cocoa, would, on a population level, be expected to reduce stroke risk by 8%, coronary artery disease mortality by 5%, and all-cause mortality by 4% [111]. Green tea appears to reduce LDL cholesterol by ≈0.2 mmol/L, which would be estimated to result in a 3% reduction in all-cause mortality and a 6% reduction in both coronary heart disease (CHD)-related mortality and total CHD events. These are profound effects and must be considered seriously in terms of the potential for dietary phytochemicals to modulate cardiovascular disease risk. To achieve the clinically important LDL reductions discussed above, 2–5 mugs green tea/d (up to one-half of the usual intake) would be required, and hence the side-effects and effectiveness of dietary regimens in reducing cardiovascular disease risk should be considered carefully [111]. On the other hand, no other effect was shown when healthy men were fed green tea catechins for three weeks (6 capsules/d providing ∼670 mg of flavanols/d) other than a slight but significant reduction in the total:HDL cholesterol ratio [112].

#### 3.1.2. Oligomeric Procyanidins

As explained above, the anti-thrombotic properties by inhibition of platelet activation as well as the platelet-dependent inflammatory response, are major factors of interest in the prevention of myocardial infarction and ischaemic heart disease, where procyanidins present in cocoa, grape extracts, and in grape seeds may exert a positive effect [113,114].

Garcia-Conesa *et al.* [115] have shown, using DNA-arrays, that apple procyanidins may change the expression of various genes *in vitro*. Human endothelial cells responded to oligomeric procyanidins by exhibiting a less migratory phenotype and by a general modulation of the expression of genes that are associated with key events in the angiogenic process. The molecular changes associated with procyanidin treatment identified in this study are consistent with the beneficial effects of flavan-3-ols on vascular function.

Using various animal models it was demonstrated that proanthocyanidins have a dose-dependent vasorelaxant effect (0.1–100 μg/mL of proanthocyanin-rich fraction from *Croton sp.*), involving the activation of the endogenous nitric oxide synthase (eNOS). This relaxant effect is endotelium dependent and involves the NO/cGMP pathway [116].

In Zucker *Fa/fa* rats taking an hyperlipidemic diet, and presenting a slight inflammatory response, it was observed that consumption of grape seed proanthocyanidins (345 mg/kg feed) produced a decrease in the levels of proinflammatory markers such as C-reactive protein (CRP), interleukin-6 (IL-6) and tumoral necrosis factor alpha (TNF-α) and an increase in the production of antiinflammatory cytokines like adiponectin [117], supporting the effects of dietary procyanidins in reducing obesity-related adipokine dysregulation to manage cardiovascular and metabolic risk factors.

Some studies in animal and *in vitro* models using different cell models have shown that the effect of dietary proanthocyanidins on plasma triglyceride levels is mediated by cholesterol 7-α-hydroxylase (CYP7A1) and nuclear receptor SHP, both implicated in lipid metabolism [118,119]. Hyperlipidemia is also one of the major risk factors for the development of cardiovascular disease. The positive effect of catechins and proanthocyanidins on blood lipidic profile in human subjects has also been reported and reviewed [108].

In human intervention trials in normo- and hypercholesterolemic subjects, the daily intake of cocoa powder (containing catechin, epicatechin, procyanidin B2, and procyanidin C1) at a dosage ≥13 g/d for four weeks had favourable effects on LDL and HDL cholesterol and oxidized LDL concentrations in plasma, especially in subjects with LDL cholesterol concentrations ≥3.23 mmol/L. The intake of polyphenol-rich foods, such as cocoa, tea, wine, fruit, and vegetables should lead to a decrease in the incidence of atherosclerotic disease [120].

In patients at high risk of CVD, the intake of cocoa polyphenols (mainly catechins and procyanidins) has shown to modulate inflammatory mediators, *i.e.*, decreasing the expression of VLA-4, CD40, and CD36 in monocytes and the serum levels of soluble endothelium-derived adhesion molecules P-selectin and intercellular adhesion molecule-1. These antiinflammatory effects may contribute to the overall benefits of cocoa consumption against atherosclerosis [121].

Studies done using human lymphocytes have shown that oligomeric procyanidins (between two and five epicatechin units) are potent stimulators of both, the innate immune system and the adaptive immunity [122]. If cocoa flavanols and procyanidins could augment the expression of inmune cells *in vivo*, their consumption would be associated with a more efficient and rapid response to immune challenges.

Lately, Modrianský and Gabrielová [9], have proposed a very interesting approach to the understanding of polyphenols cardioportective effect. As they stated, most flavonoids have an acidic character and may act as chemical uncouplers in the mitochondria, causing a higher resting energy expenditure which appears to increase cell longevity. Because flavonoids have limited bioavailability their levels in the myocardium may be low but durable in case their consumption is steady, and in this way they may have a mild uncoupling effect turning the mitochondrial inefficiency into an advantage and protecting cardiomyocytes against ischemia reperfusion injury.

Looking into the interaction with other diseases, *in vitro* data using catechin and proanthocyanidins have shown also protective effect on myocardial cells against the toxicity originated by the administration of anticancer drugs like doxorubicin [123].

### 3.2. Anthocyanins

An investigation on the promotion of an optimal platelet function *via* the reduction of platelet hyper-reactivity using dietary solutions, assayed the anti-platelet activity of physiologically relevant concentrations of the anthocyanins delphinidin-3-*O*-rutinoside, cyanidin-3-*O*-glucoside, cyanidin-3-*O*-rutinoside, and malvidin-3-*O*-glucoside, and their putative colonic metabolites, dihydroferulic acid, 3-(3-hydroxyphenyl)propionic acid, 3-hydroxyphenylacetic acid and 3-methoxy-4-hydroxypheylacetic acid, both separately and in combination. Anti-thrombotic properties were exhibited by 10 μmol/L dihydroferulic acid, and 3-(3-hydroxyphenyl)propionic acid, 1 μmol/L delphinidin-3-*O*-rutinoside, and a mixture of all the tested compounds [124]. Anthocyanins also inhibit the thrombin receptor activating peptide (TRAP)-induced platelet aggregation but did not influence platelet reactivity when faced with strong agonists such as collagen and ADP [124].

Anthocyanins are capable of acting on different cells involved in the development of atherosclerosis. The chemokine monocyte chemotactic protein 1 (MCP-1) is known to mediate in the recruitment of macrophages to sites of infection or inflammation, and direct involvement of MCP-1 on atherogenesis has been established. Anthocyanins have been shown to have a protective effect against TNF-α induced MCP-1 secretion in primary human endothelial cells [125].

Vascular endothelial growth factor (VEGF) is a major pro-angiogenic and pro-atherosclerotic factor. Anthocyanins, specifically delphinidin and cyanidin, have been shown to prevent the expression of VEGF stimulated by platelet derived growth factor(AB) (PDGF(AB) in vascular smooth muscle cells by preventing activation of p38 mitogen-activated protein kinases (p38 MAPK) and c-Jun N-terminal kinase (JNK) [126].

Anthocyanin extracts from chokeberry, bilberry, and elderberry have shown endothelium-dependent relaxation capacity in porcine coronary arteries [127]. Moreover, chronic ingestion of anthocyanins increased cardiac glutathione concentrations in rats [128]. In a recent study, rats treated with isoproterenol to induce postinfarction remodeling were fed with an alcohol-free red wine showing a protective effect on hearts by repressing hypertrophy-associated increased phosphorylation of protein kinase C (PKC) α/β II and by activating Akt/protein kinase B (Akt) [129].

Anthocyanins have an effect on cholesterol distribution, protecting endothelial cells from CD40-induced proinflammatory signalling [130].

Pelargonidin inhibits iNOS protein and mRNA expression as well as the NO production in a dose-dependent manner in macrophages exposed to an inflammatory stimulus (lipopolysaccharide, LPS). Pelargonidin also inhibits the activation of NF-κB, which is a significant transcription factor for iNOS [131]. In macrophages, blackberry anthocyanins inhibit LPS induced nitric oxide biosynthesis [132].

Ischemia/reperfusion injury leads to irreplaceable myocyte cell necrosis and apoptosis with concomitant activation of signal transducers and activators of transcription 1 (STAT1) and STAT3. It has been shown that the anthocyanin delphinidin decreases the extent of both necrotic and apoptotic cell death in cultured cardiomyocytes and reduces infarct size after ischemia in rats and that both effects are mediated by inhibition of STAT1 activation [133]. In rats, a long-term dietary intake of corn-derived anthocyanins, mainly cyanidin and pelargonidin based, made the myocardium less susceptible to ischemia-reperfusion injury *ex vivo* as well as *in vivo* when comparing it with a control consisting of anthocyanin-free corn. However results from human trials are controversial. Recently Curtis *et al.* [134], showed no effect on biomarkers of cardiovascular disease (CVD), including inflammatory biomarkers, platelet reactivity, lipids, and glucose; and liver and kidney function, as well as anthropometric measures, blood pressure, and pulse after a 12-week intervention with 500 mg cyanidin in postmenopausal women.

Many phenolic compunds are potent effectors of biologic processes and have the capacity to influence disease risk *via* several complementary and overlapping mechanisms. The current knowledge on mechanisms by which dietary phenolic compounds play a role in preventing degenerative pathologies was summarized recently [8]. In particular, the complex interactions between these dietary molecules and their molecular targets including the cell signaling pathways and response: NF-κB signaling pathway, Activator protein 1(AP-1), the Phase II enzyme activation and Nrf, and the mitogen-activated protein kinase (MAPK) signaling pathway. Future studies should focus on commonly consumed anthocyanins, in order to examine dose-response effects, and be of long enough duration to allow assessment of clinically relevant endpoints [111].

## 4. Conclusions

The available bibliographical data on the structure, distribution and bioavailability of flavanols and anthocyanins, and their role in the modulation or reduction of risk factors and the prevention of cardiovascular health problems through different aspects of bioeficacy in vascular health (platelet agregation, atherosclerosis, blood pressure, antioxidant status, inflammation-related markers, *etc.*), myocardial conditions, and whole-body metabolism (serum biochemistry, lipid profile) are consistent. The available published intervention trials provide an important snapshot of the current state of the art and facilitate the identification of future research priorities. Anthocyanins seem to have a clear effect on endothelial function and myocardium protection, even if most results come from *in vitro* studies.

In general more intervention studies in healthy subjects and in subjects at risk of cardiovascular disease or related pathologies are needed. As it has been reviewed, the metabolism and distribution of catechin and procyanidins, and more markedly of anthocyanins, is still not clarified. In order to improve the knowledge on their bioavailability and bioefficacy further studies are warranted involving human volunteers in large scale and long term trials, using different approaches (randomized, placebo-controlled studies), to better estimate the actual concentration of flavanol and anthocyanin metabolites in plasma within the context of a regular diet which includes a chronic ingestion of flavonoid-rich or dense foods and food products, as available in the many Mediterranean diets.

In the case of anthocyanidins, bioavailability studies which include intestinal flora metabolites are clearly needed in order to better understand anthocyanin bioactivity. Moreover, *in vitro* and *in vivo* studies using anthocyanins are difficult to standardize due to the chemical instability of this group of compounds and generally a good control or reference is needed so the data will bring the research to the right conclusions.

It should also be taken into account that, in general, flavonoid subclasses are present simultaneously in foods of plant origin and thus it could be difficult to establish which of the family of compounds present in that given food or food product is responsible for the potential biological effect. Therefore, it is of great interest and necessity finding reliable and efficient biomarkers of the flavanol and anthocyanin ingestion to demonstrate further biological actions or bioefficacy in CVDs.

## Figures and Tables

**Table 1.** Flavanols in fruits and berries.

**Fruit**	**Flavanols (mg/100 g fresh weight)**	**References**
**Apple**	0.1–45	[13,14,17,20]
**Apricot**	0.3–11	[13,14]
**Avocado**	0.1–0.6	[13,14]
**Banana**	0.1–10.3	[13,25]
**Black currant**	1.2	[14]
**Blackberry**	3.3–23.8	[13–15,17]
**Blueberry**	1–7	[13–15]
**Cherry**	6.3–23	[13–15,17]
**Custard apple**	18–25	[13]
**Fig**	0.1–4.8	[13,16,17]
**Grape**	0.1–20	[13–15,23,24]
**Kiwi**	0.3–0.8	[13,14]
**Loquat fruit**	2.5–2.9	[13]
**Mango**	1.7	[13]
**Peach**	2–17	[13,14]
**Pear**	0.4–12	[13,14,22]
**Persimmon**	0.4–1.7	[13,18]
**Plum**	3.7–79	[13,14,17]
**Pomegranate**	0.8–1.2	[13]
**Quince**	3–7	[13,14]
**Raspberry**	2–48	[13–15,17]
**Red currant**	2–7	[13,14]
**Strawberry**	2–6	[13–15,17]
**Strawberry tree fruit**	10–29	[13,19]

**Table 2.** Anthocyanin contents in foods of plant origin.

**Food**	**Content**	Unit *	**References**
**Apple**	0.0–60.0	mg per 100 g f.w.	[50,51]
**Bilberry**	300–698	mg per 100 g f.w.	[50,52]
**Black bean**	24.1–44.5	mg per 100 g f.w.	[53]
**Black currant**	130–476	mg per 100 g f.w.	[54,55]
**Black olives**	42–228	mg per 100 g f.w.	[52]
**Black rice**	10–493	mg per 100 g f.w.	[56]
**Blackberry**	82.5–325.9	mg per 100 g f.w.	[57,58]
**Blueberry**	61.8–299.6	mg per 100 g f.w.	[59,60]
**Bog whortleberry**	154	mg per 100 g f.w.	[50]
**Cherry**	2–450	mg per 100 g f.w.	[52,61]
**Chokeberry**	410–1480	mg per 100 g f.w.	[50,55]
**Cranberry**	67–140	mg per 100 g f.w.	[50,55]
**Crowberry**	360	mg per 100 g f.w.	[59]
**Eggplant**	8–85	mg per 100 g f.w.	[50,55]
**Elderberry**	664–1816	mg per 100 g f.w.	[62]
**Goji**	49.4	mg per 100 g f.w.	[63]
**Gooseberry**	2.0–43.3	mg per 100 g f.w.	[55,64]
**Grapefruit**	5.9	mg per 100 g f.w.	[50]
**Lettuce**	2.5–5.2	mg per 100 g f.w.	[50,55]
**Nectarine**	2.4	mg per 100 g f.w.	[50]
**Peach**	4.2	mg per 100 g f.w.	[50]
**Pear**	5–10	mg per 100 g f.w.	[52]
**Plum**	2–25	mg per 100 g f.w.	[55]
**Pomegranate, unprocessed juice**	15–252	mg per L	[65]
**Purple corn**	1642	mg per 100 g f.w.	[66]
**Raspberry**	20–687	mg per 100 g f.w.	[55,58]
**Red cabbage**	322	mg per 100 g f.w.	[55]
**Red currant**	22	mg per 100 g f.w.	[50]
**Red grape**	30–750	mg per 100 g f.w.	[67]
**Red onion, processed**	23.3–48.5	mg per 100 g f.w.	[55,68]
**Red radish**	100–154	mg per 100 g f.w.	[55,69]
**Red wine**	16.4–35	mg per 100 mL	[70–72]
**Rhubarb**	4–200	mg per 100 g f.w.	[50]
**Rowanberry**	14	mg per 100 g f.w.	[50]
**Sakatoon berry**	234	mg per 100 g f.w.	[50]
**Strawberry**	19–55	mg per 100 g f.w.	[73]

* Units expressed according to the data in the cited reference.
